# The pediatric NAFLD fibrosis index: a predictor of liver fibrosis in children with non-alcoholic fatty liver disease

**DOI:** 10.1186/1741-7015-7-21

**Published:** 2009-05-01

**Authors:** Valerio Nobili, Anna Alisi, Andrea Vania, Claudio Tiribelli, Andrea Pietrobattista, Giorgio Bedogni

**Affiliations:** 1 Department of Hepatogastroenterology and Nutrition, Pediatric Hospital IRCCS 'Bambino Gesù', Via S. Onofrio 4, 00165 Rome, Italy; 2 Center of Dietetics and Nutrition, Pediatric Clinic, 'La Sapienza' University, Viale Regina Elena 324, 00100 Rome, Italy; 3 Liver Research Center, Building Q, AREA Science Park, Strada Statale 14/km 163.5, 34012 Basovizza, Trieste, Italy

## Abstract

**Background:**

Liver fibrosis is a stage of non-alcoholic fatty liver disease (NAFLD) which is responsible for liver-related morbidity and mortality in adults. Accordingly, the search for non-invasive markers of liver fibrosis has been the subject of intensive efforts in adults with NAFLD. Here, we developed a simple algorithm for the prediction of liver fibrosis in children with NAFLD followed at a tertiary care center.

**Methods:**

The study included 136 male and 67 female children with NAFLD aged 3.3 to 18.0 years; 141 (69%) of them had fibrosis at liver biopsy. On the basis of biological plausibility, readily availability and evidence from adult studies, we evaluated the following potential predictors of liver fibrosis at bootstrapped stepwise logistic regression: gender, age, body mass index, waist circumference, alanine transaminase, aspartate transaminase, gamma-glutamyl-transferase, albumin, prothrombin time, glucose, insulin, triglycerides and cholesterol. A final model was developed using bootstrapped logistic regression with bias-correction. We used this model to develop the 'pediatric NAFLD fibrosis index' (PNFI), which varies between 0 and 10.

**Results:**

The final model was based on age, waist circumference and triglycerides and had a area under the receiver operating characteristic curve of 0.85 (95% bootstrapped confidence interval (CI) with bias correction 0.80 to 0.90) for the prediction of liver fibrosis. A PNFI ≥ 9 (positive likelihood ratio = 28.6, 95% CI 4.0 to 201.0; positive predictive value = 98.5, 95% CI 91.8 to 100.0) could be used to rule in liver fibrosis without performing liver biopsy.

**Conclusion:**

PNFI may help clinicians to predict liver fibrosis in children with NAFLD, but external validation is needed before it can be employed for this purpose.

## Background

Pediatric non-alcoholic fatty liver disease (NAFLD) is a highly prevalent and potentially serious complication of childhood obesity [1]. NAFLD encompasses a spectrum of disease from simple steatosis to non-alcoholic steatohepatitis (NASH), to fibrosis and ultimately cirrhosis [2]. While simple steatosis has a benign prognosis, NASH and fibrosis are responsible for liver-related morbidity and mortality in adults [3-6].

Although the natural history of pediatric NAFLD is not known, children with NAFLD followed at tertiary care centers do often show some degree of fibrosis and less-frequently frank cirrhosis [1,7]. The early identification of fibrosis is especially important in children because it may help to prevent the development of liver disease during adulthood [1,8].

The diagnosis of liver fibrosis is based on liver biopsy [2,9]. However, liver biopsy is invasive and limited by hazard and discomfort to the patient [10]. Thus, there is a recognized need for less-invasive strategies to identify the minority of NAFLD patients with liver fibrosis [11,12]. These considerations are even more relevant for pediatric age, where liver biopsy is generally perceived as bearing greater risk and is less acceptable than in adults [13].

Several efforts have been made in recent years to identify non-invasive markers of fibrosis in adults with NAFLD [11,14]. Studies have focused on the use of clinical features and routine laboratory exams or less readily available serum markers of liver fibrosis [11,12,14,15]. However, all studies so far have been performed in adults and there is a clear need to evaluate non-invasive approaches in children [1].

The aim of the present study was to evaluate the ability of selected clinical features and laboratory exams to predict liver fibrosis in children with NAFLD using liver biopsy as the gold standard. The choice of potential predictors was made on the basis of biological plausibility, readily availability and evidence from adult studies [11,14].

## Methods

### Study design

We studied 203 children with NAFLD consecutively admitted at the Liver Unit of the 'Bambino Gesù' Hospital (Roma, Italy) between June 2004 and April 2008. NAFLD was operationally defined as diffusely hyperechogenic liver at ultrasonography with persistently elevated (i.e. >40 U/L for at least 6 months) aspartate transaminase (AST) or alanine transaminase (ALT) after exclusion of viral, alcohol-induced, drug-induced, cholestatic and genetic causes of liver disease [8]. The probability of a child undergoing liver biopsy in this study reflected the current practice at our center, which is based mostly on the consensus of experts because of the scarcity of liver biopsy data in children [1]. The study protocol conformed to the Declaration of Helsinki and was approved by the local ethical committee. Informed consent was obtained from each patient or a responsible guardian.

### Anthropometric assessment

Weight and height were measured using standard procedures [16]. Waist circumference was measured at the highest point of the iliac crest [17,18]. Body mass index (BMI) was calculated as weight (kg)/height (m)^2^. Standard deviation scores (SDS) of weight, height and BMI were calculated from US reference data [19].

### Laboratory assessment

ALT, AST, gamma-glutamyl-transferase (GGT), albumin, prothrombin time (international normalized ratio (INR)), glucose, triglycerides and cholesterol were evaluated using standard laboratory methods. Insulin was measured by radio-immuno-assay (Myria Technogenetics, Milan, Italy). Glucose and insulin were measured at 0 (fasting), 30, 60, 90 and 120 minutes during an oral glucose tolerance test (OGTT) performed with 1.75 g glucose per kilogram of body weight (up to 75 g) [20]. The homeostasis model assessment index of insulin resistance (HOMA-IR) and the insulin sensitivity index (ISI) were calculated as surrogate markers of insulin sensitivity [21,22].

### Liver ultrasonography

Liver ultrasonography was performed by the same radiologist using a Siemens Sonoline Omnia instrument with a 5 MHz transducer (Siemens Medical Solutions, Mountain View, CA, USA). Fatty liver was diagnosed according to standard criteria [23,24].

### Liver biopsy

Liver biopsy was performed using an automatic core biopsy device (Biopince, Amedic, Sweden) equipped with a 18-G needle. Only samples with length at least 15 mm and including at least five portal tracts were considered. Liver biopsies were routinely stained with hematoxylin-eosin, Van Gieson, PAS-D and Prussian blue. The degree of fibrosis was scored according to the Nonalcoholic Steatohepatitis Clinical Research Network (0 = absence of fibrosis; 1 = perisinusoidal or portal fibrosis; 2 = perisinusoidal and portal/periportal fibrosis; 3 = septal or bridging fibrosis; 4 = cirrhosis) [9,18]. All liver biopsies were evaluated by the same pathologist who was blinded to the ultrasonography and laboratory data.

### Statistical analysis

Continuous variables are given as the median and interquartile range (IQR) owing to skewed distributions. IQR was calculated as the difference between the 75th and 25th percentile. Comparisons of continuous variables between children with and without liver fibrosis were performed with the Wilcoxon-Mann-Whitney test and those of categorical variables with the Fisher's exact test. Spearman's rho was used to perform correlation analysis. The outcome variable of prediction models was liver fibrosis of any degree (1 = yes; 0 = no). On the basis of biological plausibility, readily availability and evidence from adult studies [11,12,14,15], we evaluated the following series of potential predictors: gender, age, BMI, waist circumference, ALT, AST, GGT, albumin, INR, glucose, insulin, triglycerides and cholesterol (Model 1). Owing to the pivotal role of insulin resistance in the pathogenesis of NAFLD [1,25,26], we also evaluated two models with HOMA-IR (Model 2) or ISI (Model 3) in place of fasting glucose and insulin. To identify candidate predictors of fibrosis, we performed a stepwise logistic regression analysis on 1,000 bootstrap samples of 203 subjects (probability to enter = 0.05; probability to remove = 0.10) [27-29]. All variables apart from gender were evaluated as continuous [30]. Linearity of logits was ascertained using the Box-Tidwell procedure at preliminary univariable analysis. To linearize logits, age, ALT, AST, insulin and triglycerides were transformed using natural logarithms (log_e_). Multivariable fractional polynomials were used to test whether the fit of the multivariable models could be improved by transformation of continuous predictors [31]. As there was no gain in fit, we left the predictors untransformed in the final model. The 95% CIs of the regression coefficients of the final model were calculated using 1,000 bootstrap samples of 203 subjects with bias correction [32]. The goodness of fit of the final model was checked using standard diagnostic plots and the Hosmer-Lemeshow statistic [33]. The ability of the final model to discriminate liver fibrosis was assessed by calculating the non-parametric area under the receiver-operating characteristic curve (AUROC) with 95% CIs calculated on 1,000 bootstrap samples with bias correction [34]. The probabilities obtained from the final model were multiplied by 10 to obtain the "pediatric NAFLD fibrosis index" (PNFI). The true positive rate (TPR), true negative rate (TNR), positive likelihood ratio (PLR), negative likelihood ratio (NLR), positive predictive value (PPV) and negative predictive value (NPV) of nine PNFI cut-points were calculated [27]. All statistical tests were two-tailed and statistical significance was set to a *p*-value *p *< 0.05. Statistical analysis was performed using STATA 10.1 (StataCorp, College Station, Texas, USA).

## Results

141 (69%) out of 203 children with NAFLD had liver fibrosis. Most children had stage 1 (*n *= 115), a few had stage 2 (*n *= 9) or 3 (*n *= 17), and none had stage 4 fibrosis.

Table 1 gives the measurements of the children with and without liver fibrosis. Waist circumference (*p *< 0.0001), GGT (*p *= 0.031), triglycerides (*p *< 0.0001) and cholesterol (*p *< 0.0001) were higher in children with fibrosis than in those without fibrosis while all of the other measurements were similar.

**Table 1:** Measurements of children with and without fibrosis.

	**Fibrosis** **(*n *= 141)**	**No fibrosis** **(*n *= 62)**	*p*-value*
Gender (male/female)	96/45	40/22	0.630
Age (years)	12.1 (3.6)	11.6 (3.3)	0.807
Weight (kg)	62.0 (27.6)	58.9 (19.5)	0.378
Weight (SDS)	1.8 (1.1)	1.8 (1.3)	0.430
Height (m)	1.53 (0.23)	1.52 (0.15)	0.983
Height (SDS)	0.2 (1.4)	0.2 (2.1)	0.319
BMI (kg/m^2^)	26.4 (6.5)	25.4 (4.2)	0.078
BMI (SDS)	1.9 (0.7)	1.7 (0.6)	0.079
Waist circumference (cm)	94 (11)	87 (12)	< 0.001
ALT (U/L)	67 (82)	66 (27)	0.414
AST (U/L)	47 (32)	42 (16)	0.070
GGT (U/L)	21 (19)	18 (11)	0.031
Albumin (mg/dl)	4.5 (0.4)	4.4 (0.6)	0.175
Prothrombin time (INR)	1.0 (0.2)	1.0 (0.3)	0.756
Glucose (mg/dl)	79 (11)	82 (11)	0.166
Insulin (μU/ml)	13 (10)	9 (7)	0.174
HOMA-IR	2.5 (2.1)	2.1 (1.7)	0.289
ISI	3.5 (3.0)	3.9 (2.6)	0.243
Triglycerides (mg/dl)	94 (58)	67 (39)	< 0.001
Cholesterol (mg/dl)	166 (36)	145 (47)	< 0.001

Continuous variables are given as median (interquartile range) and categorical variables as the number of subjects with the characteristic of interest. Abbreviations: SDS = standard deviation score; BMI = body mass index; ALT = alanine transaminase; AST = aspartate transaminase; GGT = gamma-glutamyl-transferase; INR = international normalized ratio; HOMA-IR = homeostasis model assessment index of insulin resistance; ISI = insulin sensitivity index.* Wilcoxon-Mann-Whitney test for continuous variables and Fisher's exact test for categorical variables.

Table 2 gives the bootstrap inclusion fraction, that is, the number of times out of 1,000 that the candidate predictors were selected at bootstrapped stepwise logistic regression in Models 1, 2 and 3. The variables selected most frequently in all models were log_e_-transformed age (100%), waist circumference (100%), and log_e_-transformed triglycerides (≥ 99.9%). Expectedly, log_e_-transformed age and waist circumference were strongly correlated (Spearman's rho = 0.79, *p *< 0.0001). This is the likely reason why age was selected as a predictor at bootstrapped multivariable analysis despite the lack of significance at univariable analysis.

**Table 2:** Selection of candidate predictors at bootstrapped stepwise logistic regression.

	**Model 1**	**Model 2**	**Model 3**
Male gender	243	241	253
Log_e _age	**1,000**	**1,000**	**1,000**
BMI	617	625	643
Waist	**1,000**	**1,000**	**1,000**
Log_e _ALT	378	362	350
Log_e _AST	281	325	341
GGT	389	363	357
Albumin	439	423	312
INR	189	226	208
Glucose	229	--	--
Log_e _insulin	286	--	--
HOMA-IR	--	218	--
ISI	--	--	300
Log_e _triglycerides	**999**	**1,000**	**1,000**
Cholesterol	233	198	161

The bootstrap inclusion fraction, i.e. the number of bootstrap samples out of 1,000 where the candidate predictors were selected, is given. Predictors selected for inclusion in the final model are marked in bold. Abbreviations: log_e _= natural logarithm; BMI = body mass index; ALT = alanine transaminase; AST = aspartate transaminase; GGT = gamma-glutamyl-transferase; INR = international normalized ratio; HOMA-IR = homeostasis model assessment index of insulin resistance; ISI = insulin sensitivity index.

The log_e_-transformed age, waist circumference and log_e_-transformed triglycerides were thus entered into a bootstrapped stepwise logistic regression model and probabilities of liver fibrosis were computed. Table 3 gives the coefficients of the final model with standard errors calculated by bootstrap analysis with bias correction. We multiplied the probabilities generated by this model per 10 to obtain the PNFI. The PNFI can be calculated in two steps as follows.

**Table 3:** The prediction model of the pediatric non-alcoholic fatty liver disease fibrosis index

	**β**	95% CI (β)*	***p*-value**
Log_e _age (years)	-6.539	-9.358 to -4.137	< 0.001
Waist (cm)	0.207	0.135 to 0.286	< 0.001
Log_e _triglycerides (mg/dl)	1.957	1.009 to 3.018	< 0.001
Intercept	-10.074	-15.838 to -5.028	< 0.001

Abbreviations: Log_e _= natural logarithm; β = regression coefficient; 95%CI (β) = 95% confidence interval for the regression coefficient; *p*-value = value of *p *for the regression coefficient. *Obtained on 1,000 bootstrap samples of 203 subjects with bias-correction.

(1) Calculation of the linear predictor:



(2) Transformation of the linear predictor into the PNFI:



Diagnostic plots and the Hosmer-Lemeshow statistic (*p *= 0.251) showed adequate model fitting. The accuracy of the prediction as detected by the AUROC was 0.85 (95% bootstrapped CI with bias correction: 0.80 to 0.90).

Table 4 gives TPR, TNR, PLR, NLR, PPV and NPV for the cut-points of PNFI. Values of PNFI = 9 could be confidently used to rule in liver fibrosis (TNR = 98.4, PLR = 28.6 and PPV = 98.5). While the TPR (95.7%) of a PNFI value less than three suggests that this cut-point may be used to rule out liver fibrosis, the NPV (75.0%) is suboptimal owing to the high pre-test probability of liver fibrosis in our series (69%).

**Table 4:** Diagnostic accuracy of the pediatric non-alcoholic fatty liver disease fibrosis index.

**Cut-point**	***n***	**%**	**TPR**	**TNR**	**PLR**	**NLR**	**PPV**	**NPV**
≥ 1	198	97.5	99.3 (96.1 to 100.0)	6.4 (1.8 to 15.7)	1.1 (1.0 to 1.1)	0.1 (0.01 to 1.0)	70.7 (63.8 to 76.9)	80.0 (28.4 to 99.5)
≥ 2	190	93.6	97.2 (92.9 to 99.2)	14.5 (6.9 to 25.8)	1.1 (1.0 to 1.3)	0.2 (0.1 to 0.6)	72.1 (65.2 to 78.4)	69.2 (38.6 to 90.9)
≥ 3	179	88.2	95.7 (91.0 to 98.4)	29.0 (18.2 to 41.9)	1.3 (1.1 to 1.6)	0.2 (0.1 to 0.3)	75.4 (68.4 to 81.5)	75.0 (53.3 to 90.2)
≥ 4	167	82.3	93.6 (88.2 to 97.0)	43.5 (31.0 to 56.7)	1.7 (1.3 to 2.1)	0.2 (0.1 to 0.3)	79.0 (72.1 to 84.9)	75.0 (57.8 to 87.9)
≥ 5	154	75.9	88.7 (82.2 to 93.4)	53.2 (40.1 to 66.0)	1.9 (1.4 to 2.5)	0.2 (0.1 to 0.4)	81.2 (74.1 to 87.0)	67.3 (52.5 to 80.1)
≥ 6	138	68.0	81.6 (74.2 to 87.6)	62.9 (49.7 to 74.8)	2.2 (1.6 to 3.1)	0.3 (0.2 to 0.4)	83.3 (76.0 to 89.1)	60.0 (47.1 to 72.0)
≥ 7	118	58.1	76.6 (68.7 to 83.3)	83.9 (72.3 to 92.0)	4.7 (2.7 to 8.4)	0.3 (0.2 to 0.4)	91.5 (85.0 to 95.9)	61.2 (50.0 to 71.6)
≥ 8	94	46.3	62.4 (53.9 to 70.4)	90.3 (80.1 to 96.4)	6.4 (3.0 to 13.9)	0.4 (0.3 to 0.5)	93.6 (86.6 to 97.6)	51.4 (41.6 to 61.1)
≥ 9	66	32.5	46.1 (37.7 to 54.7)	98.4 (91.3 to 100.0)	28.6 (4.0 to 201.0)	0.6 (0.5 to 0.6)	98.5 (91.8 to 100.0)	44.5 (36.0 to 53.3)

95% confidence intervals are given in parentheses. Abbreviations: TPR = true positive rate; TNR = true negative rate; PLR = positive likelihood ratio; NLR = negative likelihood ratio; PPV = positive predictive value; NPV = negative predictive value.

## Discussion

We developed and cross-validated the PNFI, a non-invasive index for the prediction of liver fibrosis in children with NAFLD. The PNFI, which is obtained from three very simple measures (age, waist circumference and triglycerides) could be used in place of liver biopsy to rule in liver fibrosis in children with NAFLD followed at tertiary care centers.

Waist circumference has been identified as an independent predictor of liver fibrosis in many studies of adults with NAFLD [11,14]. We have recently shown that waist circumference is a predictor of liver fibrosis independently of the other components of the metabolic syndrome, BMI and body fat [18]. Waist circumference is a surrogate measure of visceral fat and may predict the development of NAFLD in children [1,35]. A large waist circumference is also an integral part of the definition of the metabolic syndrome both in children as in adults [36,37]. Waist circumference in pediatric age must be interpreted taking age into account [36-38]. Waist circumference and age were strongly associated in our children and it was probably because of this relationship that age was selected as a predictor of liver fibrosis at bootstrapped multivariable analysis.

Hypertriglyceridemia is a strong predictor of NAFLD in the general population and in obese children [27,39,40]. Hypertriglyceridemia was also an independent predictor of liver fibrosis in a study of obese adults [41]. The present study shows that triglycerides are predictors of liver fibrosis in children with NAFLD and that their effect adds to that of waist circumference. If this finding is confirmed in further studies, liver fibrosis may be added to the metabolic complications of the hypertriglyceridemic waist phenotype [42]. In contrast to triglycerides and in agreement with studies of NAFLD performed in the general population [27], cholesterol was not a predictor of fibrosis.

Liver enzymes have not been uniformly found to predict liver fibrosis in adults with NAFLD [14]. In the present study, GGT but not ALT and AST were higher in children with fibrosis. However, none of them was a predictor of fibrosis at multivariable analysis. This confirms what is already known from studies performed in adults, that is, that liver enzymes are not reliable markers of NAFLD severity as detected by liver biopsy [43].

Also albumin and prothrombin time did not contribute to the prediction of liver fibrosis in our children. This was not unexpected because these markers of liver function are altered in patients with advanced liver disease while only few of our children had advanced fibrosis.

Hyperinsulinemia is a common feature of pediatric NAFLD and there is some evidence that it may be an independent predictor of liver fibrosis [1,25]. In the present study, however, insulin, HOMA-IR and ISI were not associated with liver fibrosis. Even if ISI is a better marker of insulin sensitivity as compared with fasting insulin and HOMA-IR [44], this evidence had no practical implication as far as the prediction of liver fibrosis is concerned.

Although this is the first study to investigate the possibility of a non-invasive diagnosis of liver fibrosis from clinical and biochemical measurements in children with NAFLD, it has some limitations. First, our series was highly selected, being made of children followed at a tertiary center specialized in pediatric liver disease. This limitation is nonetheless common to all available scoring systems for fibrosis because the decision to perform liver biopsy is usually taken in tertiary centers and this is especially true for children [11,18]. This fact implies, however, that our scoring system probably cannot be used in primary and secondary care because the 'case mix' and the probability of liver fibrosis differ in these populations [6,45,46]. Second, even if the internal cross-validation of the scoring system was quite good according to current standards, external cross-validation is needed before PNFI can be used as predictor of liver fibrosis in clinical practice or research [6,27,45,46]. Third, most of our children had moderate fibrosis so that we could not develop a predictor of severe fibrosis [15]. However, the prevalence and the degree of fibrosis in our series strictly resemble those seen in other pediatric series and there is some advantage in detecting less-severe stages of fibrosis as the prevention of chronic liver disease is concerned [1,11]. Fourth, at the high prevalence rate of fibrosis observed in our series (69%), PNFI could be used with confidence to rule in but not to rule out fibrosis.

## Conclusion

PNFI is a simple and non-invasive index based on age, waist circumference and triglycerides that could be used in place of liver biopsy to rule in liver fibrosis in children with NAFLD followed at tertiary care centers. However, before being employed for this purpose, PNFI must be cross-validated in external populations.

## Abbreviations

ALT: alanine transaminase; AST: aspartate transaminase; BMI: body mass index; CI: confidence interval; GGT: gamma-glutamyl-transferase; HOMA-IR: homeostasis model assessment index of insulin resistance; INR: international normalized ratio (prothrombin time); ISI: insulin sensitivity index; OGTT: oral glucose tolerance test; NAFLD: non-alcoholic fatty liver disease; NASH: non-alcoholic steatohepatitis; NLR: negative likelihood ratio; NPV: negative predictive value; PLR: positive likelihood ratio; PNFI: pediatric NAFLD fibrosis index; PPV: positive predictive value; TNR: true negative rate; TPR: true positive rate.

## Competing interests

The authors declare that they have no competing interests.

## Authors' contributions

VN co-designed the study, coordinated it and co-wrote the manuscript. AA performed laboratory data collection. AV helped to improve the manuscript. CT co-designed the study and gave suggestions to improve the manuscript. AP performed clinical data collection. GB co-designed the study, performed statistical analysis and co-wrote the manuscript.

## Pre-publication history

The pre-publication history for this paper can be accessed here:


